# Efficacy and safety of Janus kinase inhibitors in non-infectious inflammatory ocular diseases: a prospective cohort study from the international AIDA network registries

**DOI:** 10.3389/fmed.2024.1439338

**Published:** 2024-08-23

**Authors:** Antonio Vitale, Judith Palacios-Olid, Valeria Caggiano, Gaafar Ragab, José Hernández-Rodríguez, Laura Pelegrín, Germán Mejía-Salgado, Laura Zarate-Pinzón, Stefano Gentileschi, Jurgen Sota, Alex Fonollosa, Ester Carreño, Carla Gaggiano, Rana Hussein Amin, Alberto Balistreri, Javier Narváez, Gian Marco Tosi, Bruno Frediani, Luca Cantarini, Alejandra de-la-Torre, Claudia Fabiani

**Affiliations:** ^1^Department of Medical Sciences, Surgery and Neurosciences, Research Center of Systemic Autoinflammatory Diseases and Behçet’s Disease Clinic, University of Siena, Siena, Italy; ^2^Azienda Ospedaliero-Universitaria Senese [European Reference Network for Rare Immunodeficiency, Autoinflammatory and Autoimmune Diseases (RITA) Center], Siena, Italy; ^3^Rheumatology Department, Hospital de Bellvitge, L’Hospitalet de Llobregat, Barcelona, Spain; ^4^Rheumatology and Clinical Immunology Unit, Internal Medicine Department, Faculty of Medicine, Cairo University, Giza, Egypt; ^5^Faculty of Medicine, Newgiza University, 6th of October City, Egypt; ^6^Department of Autoimmune Diseases, Institut d’Investigacions Biomèdiques August Pi I Sunyer, Hospital Clínic of Barcelona [European Reference Network for Rare Immunodeficiency, Autoinflammatory and Autoimmune Diseases (RITA) Center], University of Barcelona, Barcelona, Spain; ^7^Neuroscience Research Group (NEUROS), NeuroVitae Center, Escuela de Medicina y Ciencias de la Salud, Universidad del Rosario, Bogotá, Colombia; ^8^Department of Ophthalmology, Biocruces Bizkaia Health Research Institute, Cruces University Hospital, University of the Basque Country, Barakaldo, Spain; ^9^Department of Ophthalmology, Hospital Universitario Rey Juan Carlos, Madrid, Spain; ^10^Department of Ophthalmology, Hospital Universitario Fundación Jiménez Díaz, Madrid, Spain; ^11^Department of Ophthalmology, Cairo University, Giza, Egypt; ^12^Bioengineering and Biomedical Data Science Lab, Department of Medical Biotechnologies, University of Siena, Siena, Italy; ^13^Ophthalmology Unit, Department of Medicine, Surgery and Neurosciences, University of Siena, Siena, Italy

**Keywords:** baricitinib, scleritis, tofacitinib, upadacitinib, uveitis

## Abstract

**Introduction:**

Non-infectious inflammatory ocular diseases pose significant challenges in diagnosis and management, often requiring systemic immunosuppressive therapy. Since Janus kinase (JAK) inhibitors may represent a novel therapeutic option for these disorders, the present study aimed to expand current knowledge about their efficacy and safety in patients with these conditions.

**Methods:**

This prospective cohort study included 12 adult patients from the international AutoInflammatory Disease Alliance (AIDA) Network registries dedicated to non-infectious ocular inflammatory conditions. We assessed ocular flares, visual acuity, disease course, and complications before and after initiating JAK inhibitor therapy.

**Results:**

Ocular inflammation was related to a systemic disease in 8 (66.7%) patients as follows: spondyloarthritis (*n* = 3), peripheral psoriatic arthritis (*n* = 1), rheumatoid arthritis (*n* = 1), antinuclear antibodies (ANA) positive juvenile idiopathic arthritis (*n* = 1), Behçet’s syndrome (*n* = 1), Vogt-Koyanagi-Harada syndrome (*n* = 1). In total, 4 patients received baricitinib, 1 patient received tofacitinib, and 7 patients underwent upadacitinib treatment. The overall average duration of JAK inhibitors treatment was 8.6 ± 5.5 months (ranging from 3 to 20 months). At the last assessment, ocular disease control was complete in 12/12 patients. One patient discontinued baricitinib due to poor compliance after a 12-month relapse-free period. The incidence of ocular flares was 125 episodes/1.000 person-months prior to the initiation of JAK inhibitors and 28.6 episodes/1.000 person-months thereafter. The incidence rate ratio for experiencing a relapse before starting a JAK inhibitor compared to the following period was 4.37 (95% CI 1.3–14.7, *p*-value: 0.02).

**Conclusion:**

JAK inhibitors demonstrate efficacy and safety in controlling ocular inflammatory relapses, confirming that they represent a valuable treatment option for patients with non-infectious inflammatory ocular diseases resistant to conventional treatments.

## Introduction

Non-infectious inflammatory ocular diseases encompass a diverse group of conditions, including uveitis, scleritis, and keratitis, that can arise either independently or as part of systemic autoimmune disorders. These disorders have the potential to lead to various vision-threatening complications, posing significant challenges in both diagnosis and management due to their varied presentations and the need for precise therapeutic strategies (1, 2).

The effective management of ocular inflammatory diseases typically involves a combination of systemic immunosuppressive therapy and loco-regional ocular treatments. Traditional approaches often utilize corticosteroids to control the acute phase, and disease-modifying antirheumatic drugs (DMARDs) for long-term management. Biologic agents, such as monoclonal tumor necrosis factor (TNF)-α inhibitors, tocilizumab, and rituximab, have demonstrated effectiveness in managing ocular inflammation (3–5). However, severe cases may remain unresponsive and fail to achieve remission (3, 4, 6).

The Janus kinase pathway plays a crucial role in regulating inflammatory cells, cytokine synthesis, and proinflammatory signal transduction. Dysregulation of this pathway is closely associated with the development of various inflammatory and autoimmune conditions. Consequently, JAK inhibitors hold promise for mitigating the inflammatory cascade typically observed in ocular inflammatory conditions (6, 7). However, the therapeutic application of JAK inhibitors, particularly in immune-mediated ocular diseases, is still relatively novel, and substantial research data on this specific area of interest remains limited. Thus, to address this gap and explore the potential of JAK inhibitors, we conducted a multicenter study aimed at assessing the efficacy and safety of JAK inhibitors in a cohort of adult patients with non-infectious inflammatory ocular diseases.

## Patients and methods

This is a prospective registry-based cohort study enrolling patients with non-infectious uveitis and scleritis receiving JAK inhibitors treatment during their adulthood. Patients’ demographic, clinical, ophthalmological and therapeutic data were drawn from the International AutoInflammatory Disease Alliance (AIDA) Network registries dedicated to uveitis, scleritis and Behçet’s syndrome (8–10).

The index date was the time at the start of one of the following JAK inhibitors: baricitinib, tofacitinib, upadacitinib. The follow-up period ranged between the start of JAK inhibitor and the last assessment into the AIDA Network inserted up to February 2024. Patients with neoplasms, infectious ocular diseases and traumatic ocular inflammation were excluded.

The primary aim of the study was to assess the effectiveness of JAK inhibitors in controlling intraocular inflammation. The secondary aim was to report the safety profile of JAK inhibitors in patients with intraocular inflammation. The primary endpoint was represented by the frequency of ocular inflammatory relapses observed during the 12 months preceding the start of JAK inhibitors and those observed during JAK inhibitor treatment. Additional endpoints testing the effectiveness of JAK inhibitors on ocular inflammation consisted of the occurrence of retinal vasculitis and/or uveitic macular edema (UME), any changes in the best-corrected visual acuity (BCVA), variations in the daily dosage of glucocorticoids as prednisone (PDN) or equivalent, and the identification of new ocular complications between the start of JAK inhibitors and the last follow-up visit. The secondary endpoint was represented by the occurrence of any adverse event during JAK inhibitors treatment.

Ocular flares and relapses were classified according to the standardized uveitis nomenclature (SUN) Working Group criteria for uveitic flares (11) and according to Sen et al. (12) classification criteria for scleritis. Chronic uveitis was defined as persistent ocular inflammation with relapse in < 3 months after discontinuing treatment; recurrent uveitis was defined as repeated episodes separated by periods of inactivity without treatment lasting > 3 months. The diagnosis of UME and active retinal vasculitis was based on clinical, optical coherence tomography, and fluorangiographic findings. BCVA was measured with Snellen chart in decimal fractions at any follow-up visit. Glucocorticoids (GCs) were analyzed as mg/day of PDN or equivalent.

The study was performed according to the tenets of the Declaration of Helsinki and was approved by the Ethics Committee of the Azienda Ospedaliero-Universitaria of Siena, Siena, Italy in June 2019 (Ref. N. 14951). All patients provided their informed consent to participate in the study at the time of the enrollment into the AIDA Network registries.

Descriptive statistics included sample sizes, percentages, frequency counts, mean, median, standard deviations and interquartile range calculations. Pairwise comparisons between the start of JAK inhibitors and the last assessment were performed by using the Student *t*-test or the Mann-Whitney U test, according to data distribution assessed through the Shapiro–Wilk test. The incidence rate of developing ocular inflammatory flares was calculated as number of ocular flares divided the total number of months of observation (person-months) both before starting the study treatment (the 12 months preceding the start of JAK inhibitors, i.e., 144 months for 12 patients) and during the study period. Later, the incidence rate ratio (IRR), the corresponding 95% confidence interval (CI) and the *p*-value were calculated by using the RStudio software, version 4.3.0, as for all the other statistics previously described. All statistics were two-sided; the level of statistical significance was set at a *p*-value of 0.05 in all statistical computations.

## Results

**FIGURE 1:**
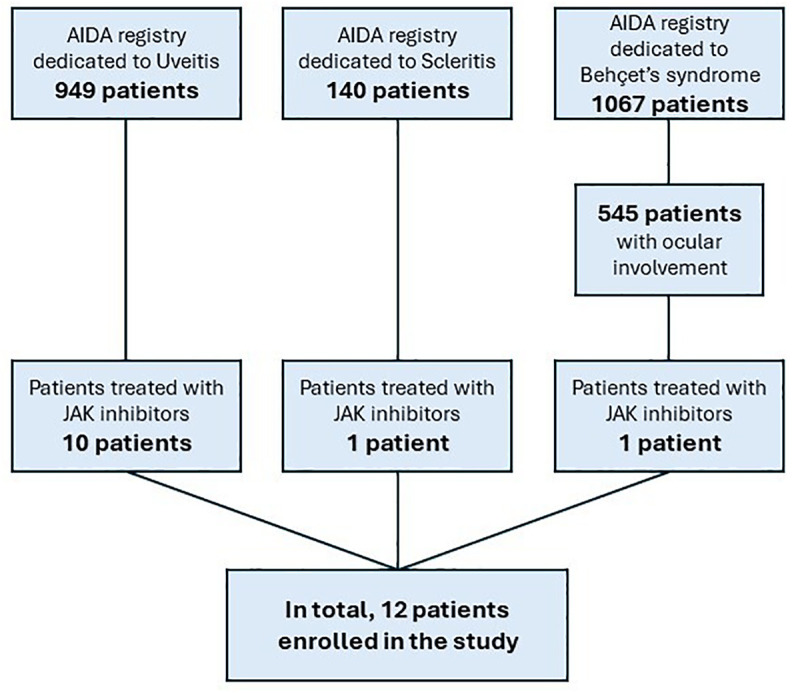
Flow-chart explaining the selection of patients included in this study starting from the total number of patients included in the AutoInflammatory Disease Alliance (AIDA) Network project.

In total, 12 patients (7 females) with non-infectious ocular inflammatory conditions were enrolled. All patients were Caucasian. Ten patients were from the International AIDA Network dedicated to uveitis (8), one from the International AIDA Network registry dedicated to scleritis (9), and one from the International AIDA Network registry dedicated to Behçet’s syndrome (10). Figure 1 showcases the flow-chart leading to the identification of patients included in this study.

Ocular inflammation was associated to a systemic disease in 8 (66.7%) patients as follows: spondyloarthritis (*n* = 3), peripheral psoriatic arthritis (*n* = 1), rheumatoid arthritis (*n* = 1), antinuclear antibodies (ANA) positive juvenile idiopathic arthritis (*n* = 1), Behçet’s syndrome (*n* = 1), and Vogt-Koyanagi-Harada syndrome (*n* = 1). One patient suffered from punctate inner choroidopathy and additional 3 patients suffered from idiopathic oculo-specific inflammation. The ocular involvement was bilateral in 6 (50%) patients and unilateral in 6 (50%) patients: as a whole, 18 eyes were involved with inflammation. The mean age at ocular disease onset was 36.6 ± 20.7 years, the mean age at diagnosis was 37.9 ± 20.7 years, the mean age at the enrollment in this study was 49.2 ± 16.1 years. The median duration of ocular inflammatory disease was 97.5 (IQR = 251) months. Specifically, there were 6 eyes affected by anterior uveitis, 1 by anterior sclerouveitis, 1 by anterior and posterior scleritis, 4 by posterior uveitis, 2 by pars planitis, and 4 by panuveitis.

In uveitic eyes, the presentation was acute in 9 eyes, insidious in 4 eyes; the remaining cases were not specified in the registry. The course of inflammatory episodes was chronic in 7 eyes and recurrent in 5 eyes; information about ocular disease course was not available for 6 eyes.

Table 1 describes the demographic, clinical and therapeutic features of the 12 patients enrolled in the study; Table 2 clarifies the treatment approaches performed prior to the start of JAK inhibitors.

**TABLE 1:** Demographic, clinical and therapeutic features describing the twelve patients enrolled.

Sex	Age at ocular disease onset, years	Age at diagnosis, years	Ocular diagnosis	Systemic or oculo-specific diagnosis	Treatment performed	Age at the start of treatment, years	Main reason for starting JAK inhibitors	Treatment duration, months	GCs at start, mg/day	GCs at last assessment, mg/day
Female	33	35	Posterior uveitis (multifocal choroiditis)	Punctate inner choroidopathy	Upadacitinib 15 mg/day plus Leflunomide 20 mg/day	38.5	Ocular activity	6	0	0
Female	51.2	51.4	Pars planitis	Idiopathic	Upadacitinib 15 mg/day	54.2	Ocular activity	20	0	0
Female	81.2	81.2	Anterior uveitis (iridocyclitis)	Idiopathic	Upadacitinib 15 mg/day plus azathioprine 200 mg/day	83.3	Ocular activity	3	Sub-tenon corticosteroids	0
Male	29.8	36.3	Anterior uveitis (iridocyclitis)	Seronegative spondyloarthritis	Upadacitinib 15 mg/day	57	Extraocular activity	3	0	0
Female	40.9	40.9	Panuveitis	Seronegative spondyloarthritis	Upadacitinib 15 mg/day	49.5	Ocular activity	6	0	0
Female	59.2	59.2	Anterior sclerouveitis	Rheumatoid arthritis	Baricitinib 2 mg/day	61	Both ocular and extraocular activity	17	PDN 15	0
Male	12.8	13.8	Panuveitis	Vogt-Koyanagi-Harada syndrome	Baricitinib 4 mg/day	19.9	Ocular activity	7	PDN 15	7.5 PDN
Female	29	29	Posterior uveitis (multifocal choroiditis)	Idiopathic	Baricitinib 4 mg/day	45.2	Ocular activity	3	PDN 30	12.5 PDN
Male	32.5	39	Anterior uveitis	Behçet’s disease	Upadacitinib 15 mg/day plus azathioprine 200 mg/day	57	Ocular activity	11	Peribulbar corticosteroid injections	0
Male	44	44	Anterior scleritis, posterior scleritis	Psoriatic arthritis	Upadacitinib 15 mg/day plus sulfasalazine 1,000 mg/day	50	Both ocular and extraocular activity	7	0	0
Female	22.6	22.6	Anterior uveitis (iridocyclitis)	Seronegative spondyloarthritis	Tofacitinib 10 mg/die	57.8	Extraocular activity	8	PDN 50	0
Male	2.5	2.6	Anterior uveitis	ANA positive juvenile idiopathic arthritis	Baricitinib 4 mg/day	26.5	Both ocular and extraocular activity	12	0	0

Each row is referred to one patient. ANA, anti-nuclear autoantibodies; GCs, glucocorticoids; JAK, Janus kinase; PDN, prednisone.

**TABLE 2:** Treatment preceding the start of Janus kinases (JAK) inhibitors.

Treatment	*N* (%)
**Glucocorticoids (GCs)**	
Topical GCs	10 (14 eyes)–83.3%
Local/regional GCs injections	2 (3 eyes)–16.7%
Systemic GCs	9 (75%)
Oral GCs	9 (75%)
Endovenous GCs	1 (8.3%)
**cDMARDs**	
Methotrexate	7 (58.3%)
Azathioprine	4 (33.33%)
Cyclosporine A	4 (33.33%)
Sulfasalazine	3 (25%)
**bDMARDs**	
Adalimumab	10 (83.3%)
Golimumab	6 (50%)
Infliximab	5 (41.7%)
Etanercept	2 (16.7%)
Certolizumab	2 (16.7%)
Tocilizumab	1 (6.25%)

bDMARDs, biologic disease modifying anti-rheumatic drugs; cDMARDs, conventional disease modifying anti-rheumatic drugs; GCs, glucocorticoids; *n*, number.

### Treatment details

The usage of JAK inhibitors in our cohort was as follows: 4 patients received baricitinib, 1 patient received tofacitinib, and 7 patients underwent upadacitinib treatment. The decision to initiate this treatment was based on ocular involvement in 7 cases, extraocular activity in 2 cases, and both ocular and extraocular involvement in 3 cases. The average duration of JAK inhibitors treatment was 8.6 ± 5.5 months (ranging from 3 to 20 months), accounting for a total follow-up duration of 103 person-months. Specifically, the average duration of upadacitinib treatment was 7.4 ± 6.2 months, baricitinib treatment lasted for 9.5 ± 4.8 months, and tofacitinib treatment was administered for 8 months.

Throughout the treatment period, 3 eyes (2 patients) experienced a single relapse each, which was effectively managed with both topical and systemic GCs, resulting in complete control of ocular disease. These relapses occurred within the three-month assessment and were considered extensions of the ocular inflammation observed at the start of JAK inhibitor therapy.

During the 12 months preceding the start of JAK inhibitors, ocular inflammatory relapses were observed in all the patients enrolled. In particular, the incidence of ocular flares was 125 episodes/1.000 person-months prior to the initiation of JAK inhibitors and decreased to 28.6 episodes/1.000 person-months thereafter; the three aforementioned relapses that occurred in the first three months were included in the JAK inhibitors treatment period. The IRR of experiencing a relapse before starting a JAK inhibitor compared to the following period was 4.37 (95% CI 1.3–14.7, *p*-value: 0.02). The number needed to treat ocular flares was 10.4.

The median BCVA was 1.0 (IQR: 0.2, range 0.03–1.0) at the start of treatment and remained at 1.0 (IQR: 0.1, range: 0.06–1.0) at the last assessment, with no statistically significant differences observed (*p*-value: 0.63).

Prior to initiating treatment with JAK inhibitors, UME had been observed in 3 patient (4 eyes) and retinal vasculitis in 1 patient (2 eyes). Conversely, no episodes of UME or retinal vasculitis occurred during treatment with JAK inhibitors.

At the start of JAK inhibitor treatment, the following ocular complications were reported in 7 patients: rhegmatogenous retinal detachment (3 eyes), steroid-induced cataract (3 eye), corneal thinning (1 eye), retinal pigment epithelial alterations (1 eye), chorioretinal scars (1 eye), chorioretinal atrophy (1 eye), posterior synechiae (1 eye), peripheral anterior synechiae (1 eye), open-angle-glaucoma (1 eye), macular atrophy (1 eye), choroidal neovascularization (1 eye) and chorioretinal scars (1 eye). At the last follow-up visit while on JAK inhibitors, no further ocular complications developed.

Regarding concomitant treatment, 3 patients (4 eyes) were on topical GCs upon initiation of JAK inhibitor therapy. After three months, 2 patients (2 eyes) continued topical treatment, one of which required bilateral peribulbar injection; one patient (2 eyes) continued the topical treatment up to the 6-month assessment. Upon initiating of JAK inhibitor therapy, 4 patients were on systemic GCs therapy with an average dose of 28.4 ± 15.7 mg of PDN or equivalent. After three months, 2 patients were still on systemic GCs therapy with an average dose of 9.4 ± 8 mg of PDN or equivalent. At the last follow-up, 2 patients were still on systemic GCs, with a dose of 7.5 and 12.5 mg/day of PDN, respectively.

Four patients were receiving combination therapy with cDMARDs at the start of treatment with JAK inhibitors: two were on azathioprine, one was on leflunomide, and one was on sulfasalazine. The follow-up period while on combination therapy was three months and eleven months for the patients on azathioprine, seven months for the patient in therapy with sulfasalazine, and six months for the patient treated with leflunomide. None of these patients discontinued treatment and there were no changes in cDMARDs dosage during JAK inhibitor treatment. Additionally, there were no new cases of cDMARDs initiation during treatment with JAK inhibitors.

At the last assessment, complete ocular disease control was achieved in 12/12 patients. All but one patient continued treatment for the whole follow-up period. In this case, poor compliance was the reason for the JAK inhibitor (baricitinib) discontinuation after a 12-month treatment period. However, this patient was followed for an additional 10-month period without experiencing any further ocular relapses. In the remaining cases, no JAK inhibitor dose tapering was observed.

As for the safety profile, only one adverse event was reported during JAK inhibitor treatment, consisting of episodes of myalgia that the patient attributed to drug intake.

## Discussion

The results obtained in this study support the effective use of JAK inhibitors in controlling non-infectious uveitis and scleritis. In particular, there was a significant reduction in the risk of experiencing ocular inflammatory recurrences when comparing relapses occurring during the 12 months prior to the initiation of JAK inhibitors with those observed during the study treatments.

Additionally, the efficacy of JAK inhibitors in managing ocular inflammatory diseases was corroborated by the absence of episodes of UME and vasculitis during the follow-up period, as well as the absence of further ocular inflammatory complications at the last assessment. This suggests the prevention of new inflammatory or treatment-induced ocular damage, further supported by the preservation of visual acuity. The effectiveness of JAK inhibitors was accompanied by an excellent safety profile in this cohort.

These results expand upon the limited published data concerning the role of JAK inhibitors in patients with inflammatory ocular diseases, which have primarily relied on case reports and small case series from pediatric patients (13, 14). In particular, a recent case series by Dutta Majumder et al. (15) reported on nine children diagnosed with uveitis and one with scleritis receiving tofacitinib treatment. Most of the patients suffered from juvenile idiopathic arthritis, with anterior uveitis being the most frequent subtype of ocular inflammation. Remission of uveitis was achieved in all but two children, with a significant steroid sparing effect and an improvement in visual acuity (15). Additionally, Miserocchi et al. (16) published a case series involving four patients with uveitis associated with juvenile idiopathic arthritis treated with either baricitinib or tofacitinib. The articular disease did not respond as effectively to JAK inhibitors, all showed improvement of uveitis defined as a reduction of anterior chamber inflammation with resolution of UME, and decreased frequency of flare-ups (16). Similarly, Paley et al. (17) reported on two patients with non-infectious uveitis and scleritis refractory to conventional steroid therapies and biologic agents including adalimumab and infliximab. Both patients added tofacitinib to methotrexate experiencing a significant improvement in ocular inflammation within four weeks of therapy initiation and maintaining disease remission for several months up to the end of the follow-up (17). Remarkable results were also observed in a case with ocular inflammation related to Vogt-Koyanagi-Harada disease and in cases with ocular surface disease related to rheumatoid arthritis (18, 19).

The results obtained from this prospective study are in line with the literature published to date and widens the evidence on the role of JAK inhibitors based on a relatively greater number of patients treated with different JAK inhibitors.

Unlike the findings reported by Dutta Majumder et al. we did not observe a statistically significant effect on either the GCs sparing effect or the BCVA improvement. Several factors may account for these differences. Firstly, in the present study, only one patient suffered from juvenile idiopathic arthritis, whereas this condition was observed in roughly half of the patients enrolled by Dutta Majumder et al. (15). Additionally, the number of eyes with ocular complications at the start of JAK inhibitors was remarkable in our study and this could have affected the improvement of visual acuity. Moreover, the shorter duration of the follow-up period in our study compared to Dutta Majumder et al. (15) could have impacted the outcomes. Furthermore, the different anatomic types of uveitis, which were more frequently anterior in the study by Dutta Majumder et al. (15) and the different ages of the two cohorts may also have played a role in the observed outcomes.

Noteworthy, while all patients achieved complete control of ocular inflammatory disease at the last assessment, two out of the four patients initially undergoing systemic GCs still required steroids at the last follow-up visit. The lack of GCs withdrawal in these two cases was either due to systemic disease activity or the short follow-up duration. Based on these data, a GCs sparing effect may be supposed when treating ocular inflammation with JAK inhibitors, but a wider number of patients are required to ascertain this aspect on a statistical threeshold. Similarly, cDMARDs concomitantly used with JAK inhibitors were only sparingly employed in this study. While this allows for a clearer analysis of the effectiveness of JAK inhibitors, combination with cDMARDs may have potentially enabled a faster onset of action and even better outcomes. Therefore, this study cannot make any assumptions about the role of combination therapy versus mono therapy; similarly, a cDMARD sparing effect cannot be evaluated.

Although this study faces with an intriguing topic, some limitations need consideration. These include the small sample size and the lack of a control group for comparisons. Additionally, the duration of follow-up may not be sufficient to fully evaluate the long-term safety and effectiveness of JAK inhibitors in our cohort. However, our results contribute to the growing preliminary evidence supporting the effectiveness of JAK inhibitors in controlling ocular inflammation, particularly in adult patients where data are lacking. Particularly, the study corroborates the potential role of these agents as a promising therapeutic option for cases refractory to conventional treatments.

In conclusion, this study provides further evidence of the potential benefit of JAK inhibitors in managing non-infectious inflammatory ocular diseases. Nevertheless, further studies are needed to confirm these findings in the long-term and establish the optimal role of JAK inhibitors in the treatment of these sight-threatening conditions.

## Data Availability

The data analyzed in this study is subject to the following licenses/restrictions: data will be available upon reasonable request to the corresponding author. Requests to access these datasets should be directed to cantarini@unisi.it.
