# Corrigendum: Efficacy and safety of Janus kinase inhibitors in non-infectious inflammatory ocular diseases: a prospective cohort study from the international AIDA network registries

**DOI:** 10.3389/fmed.2024.1489092

**Published:** 2024-09-13

**Authors:** Antonio Vitale, Judith Palacios-Olid, Valeria Caggiano, Gaafar Ragab, José Hernández-Rodríguez, Laura Pelegrín, Germán Mejía-Salgado, Laura Zarate-Pinzón, Stefano Gentileschi, Jurgen Sota, Alex Fonollosa, Ester Carreño, Carla Gaggiano, Rana Hussein Amin, Alberto Balistreri, Javier Narváez, Gian Marco Tosi, Bruno Frediani, Luca Cantarini, Alejandra de-la-Torre, Claudia Fabiani

**Affiliations:** ^1^Department of Medical Sciences, Surgery and Neurosciences, Research Center of Systemic Autoinflammatory Diseases and Behçet's Disease Clinic, University of Siena, Siena, Italy; ^2^Azienda Ospedaliero-Universitaria Senese [European Reference Network for Rare Immunodeficiency, Autoinflammatory and Autoimmune Diseases (RITA) Center], Siena, Italy; ^3^Rheumatology Department, Hospital de Bellvitge, L'Hospitalet de Llobregat, Barcelona, Spain; ^4^Rheumatology and Clinical Immunology Unit, Internal Medicine Department, Faculty of Medicine, Cairo University, Giza, Egypt; ^5^Faculty of Medicine, Newgiza University, 6th of October City, Egypt; ^6^Department of Autoimmune Diseases, Institut d'Investigacions Biomèdiques August Pi I Sunyer, Hospital Clínic of Barcelona [European Reference Network for Rare Immunodeficiency, Autoinflammatory and Autoimmune Diseases (RITA) Center], University of Barcelona, Barcelona, Spain; ^7^Neuroscience Research Group (NEUROS), NeuroVitae Center, Escuela de Medicina y Ciencias de la Salud, Universidad del Rosario, Bogotá, Colombia; ^8^Department of Ophthalmology, Biocruces Bizkaia Health Research Institute, Cruces University Hospital, University of the Basque Country, Barakaldo, Spain; ^9^Department of Ophthalmology, Hospital Universitario Rey Juan Carlos, Madrid, Spain; ^10^Department of Ophthalmology, Hospital Universitario Fundación Jiménez Díaz, Madrid, Spain; ^11^Department of Ophthalmology, Cairo University, Giza, Egypt; ^12^Bioengineering and Biomedical Data Science Lab, Department of Medical Biotechnologies, University of Siena, Siena, Italy; ^13^Ophthalmology Unit, Department of Medicine, Surgery and Neurosciences, University of Siena, Siena, Italy

**Keywords:** baricitinib, scleritis, tofacitinib, upadacitinib, uveitis

In the published article, there was an error in Figure 1 as published. In the Figure 1 there are several errors in the panels, which are crucial for the selection of patients included in the study. “Uveitis registry” was changed to “AIDA registry”; “Behçet's disease” was removed from the middle right hand side panel; “Behçet's disease patients with ocular involvement treated with JAK inhibitors” was changed to “Patients treated with JAK inhibitors” in the bottom right hand side panel. The corrected Figure 1 and its caption appear below.

**Figure 1:**
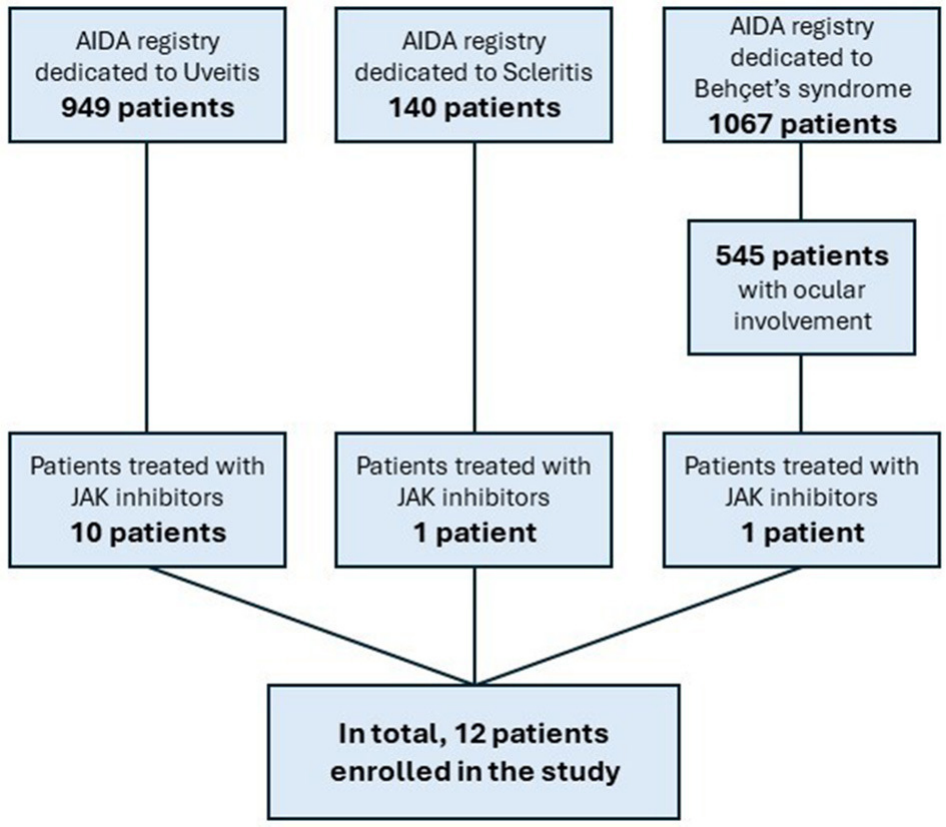
Flow-chart explaining the selection of patients included in this study starting from the total number of patients included in the AutoInflammatory Disease Alliance (AIDA) Network project.

In the published article, there was an error in Table 1 as published. In Table 1, there is a typographical error in the second to last row, “Extra ocular” was changed to “Extraocular”. The corrected Table 1 and its caption appear below.

**Table 1:** Demographic, clinical and therapeutic features describing the twelve patients enrolled.

**Sex**	**Age at ocular disease onset, years**	**Age at diagnosis, years**	**Ocular diagnosis**	**Systemic or oculo-specific diagnosis**	**Treatment performed**	**Age at the start of treatment, years**	**Main reason for starting JAK inhibitors**	**Treatment duration, months**	**GCs at start, mg/day**	**GCs at last assessment, mg/day**
Female	33	35	Posterior uveitis (multifocal choroiditis)	Punctate inner choroidopathy	Upadacitinib 15 mg/day plus leflunomide 20 mg/day	38.5	Ocular activity	6	0	0
Female	51.2	51.4	Pars planitis	Idiopathic	Upadacitinib 15 mg/day	54.2	Ocular activity	20	0	0
Female	81.2	81.2	Anterior uveitis (iridocyclitis)	Idiopathic	Upadacitinib 15 mg/day plus azathioprine 200 mg/day	83.3	Ocular activity	3	Sub-tenon corticosteroids	0
Male	29.8	36.3	Anterior uveitis (iridocyclitis)	Seronegative spondyloarthritis	Upadacitinib 15 mg/day	57	Extraocular activity	3	0	0
Female	40.9	40.9	Panuveitis	Seronegative spondyloarthritis	Upadacitinib 15 mg/day	49.5	Ocular activity	6	0	0
Female	59.2	59.2	Anterior sclerouveitis	Rheumatoid arthritis	Baricitinib 2 mg/day	61	Both ocular and extraocular activity	17	PDN 15	0
Male	12.8	13.8	Panuveitis	Vogt-Koyanagi-Harada syndrome	Baricitinib 4 mg/day	19.9	Ocular activity	7	PDN 15	7.5 PDN
Female	29	29	Posterior uveitis (multifocal choroiditis)	Idiopathic	Baricitinib 4 mg/day	45.2	Ocular activity	3	PDN 30	12.5 PDN
Male	32.5	39	Anterior uveitis	Behçet's syndrome	Upadacitinib 15 mg/day plus azathioprine 200 mg/day	57	Ocular activity	11	Peribulbar corticosteroid injections	0
Male	44	44	Anterior scleritis, posterior scleritis	Psoriatic arthritis	Upadacitinib 15 mg/day plus sulfasalazine 1,000 mg/day	50	Both ocular and extraocular activity	7	0	0
Female	22.6	22.6	Anterior uveitis (iridocyclitis)	Seronegative spondyloarthritis	Tofacitinib 10 mg/die	57.8	Extraocular activity	8	PDN 50	0
Male	2.5	2.6	Anterior uveitis	ANA positive juvenile idiopathic arthritis	Baricitinib 4 mg/day	26.5	Both ocular and extraocular activity	12	0	0

Each row is referred to one patient.

ANA, anti-nuclear autoantibodies; GCs, glucocorticoids; JAK, Janus Kinase; PDN, prednisone.

In the published article, there was an error. There are four sentences in which there are either structural or typographical errors.

A correction has been made to **Patients and methods**, Paragraph 1. This sentence previously stated:

“international AutoInflammatory Disease Alliance (AIDA) Network registries dedicated to uveitis, scleritis and Behçet's disease”.

The corrected sentence appears below:

“International AutoInflammatory Disease Alliance (AIDA) Network registries dedicated to uveitis, scleritis and Behçet's syndrome”.

A correction has been made to **Results**, Paragraph 1. This sentence previously stated:

“and one from the International AIDA Network registry dedicated to Behçet's disease (10)”.

The corrected sentence appears below:

“and one from the International AIDA Network registry dedicated to Behçet's syndrome (10)”.

A correction has been made to **Treatment details**, Paragraph 8. This sentence previously stated:

“Four patients were receiving combination therapy with cDMARDs at the start of treatment with JAK inhibitors: two were on azathioprine, and one on sulfasalazine. The follow-up period while on combination therapy was three months and eleven months for the patients on azathioprine, and seven months for the patient treated with sulfasalazine”.

The corrected sentence appears below:

“Four patients were receiving combination therapy with cDMARDs at the start of treatment with JAK inhibitors: two were on azathioprine, one was on leflunomide, and one was on sulfasalazine. The follow-up period while on combination therapy was three months and eleven months for the patients on azathioprine, seven months for the patient in therapy with sulfasalazine, and six months for the patient treated with leflunomide”.

A correction has been made to **Discussion**, Paragraph 6. This sentence previously stated:

“The lack of Gas withdrawal in these two cases was either due to systemic disease activity or the short follow-up duration”.

The corrected sentence appears below:

“The lack of GCs withdrawal in these two cases was either due to systemic disease activity or the short follow-up duration”.

The authors apologize for this error and state that this does not change the scientific conclusions of the article in any way. The original article has been updated.

